# Effects of RIPC on the Metabolomical Profile during Lower Limb Digital Subtraction Angiography: A Randomized Controlled Trial

**DOI:** 10.3390/metabo13070856

**Published:** 2023-07-18

**Authors:** Karl Kuusik, Teele Kasepalu, Mihkel Zilmer, Jaan Eha, Kaido Paapstel, Kalle Kilk, Aune Rehema, Jaak Kals

**Affiliations:** 1 Department of Cardiology, Institute of Clinical Medicine, University of Tartu, Puusepa 8, 50406 Tartu, Estonia; teele.kasepalu@kliinikum.ee (T.K.); jaan.eha@kliinikum.ee (J.E.); kaido.paapstel@kliinikum.ee (K.P.); 2 Heart Clinic, Tartu University Hospital, Puusepa 8, 50406 Tartu, Estonia; 3 Department of Biochemistry, Institute of Biomedicine and Translational Medicine, University of Tartu, Puusepa 8, 50406 Tartu, Estonia; mihkel.zilmer@ut.ee (M.Z.); kalle.kilk@ut.ee (K.K.); aune.rehema@ut.ee (A.R.); jaak.kals@kliinikum.ee (J.K.); 4 Department of Surgery, Institute of Clinical Medicine, University of Tartu, Puusepa 8, 50406 Tartu, Estonia; 5 Department of Vascular Surgery, Surgery Clinic, Tartu University Hospital, Puusepa 8, 50406 Tartu, Estonia

**Keywords:** remote ischemic preconditioning, lower extremity arterial disease, metabolomics, digital subtraction angiography, percutaneous transluminal angioplasty, glutamate, taurine, lyso-phosphatidylcholines, asymmetric dimethyl arginine, arginine

## Abstract

Remote ischemic preconditioning (RIPC) has demonstrated protective effects in patients with lower extremity arterial disease (LEAD) undergoing digital subtraction angiography (DSA) and/or percutaneous transluminal angioplasty (PTA). This study aimed to investigate the impact of RIPC on the metabolomical profile of LEAD patients undergoing these procedures and to elucidate its potential underlying mechanisms. A total of 100 LEAD patients were enrolled and randomly assigned to either the RIPC group (n = 46) or the sham group (n = 54). Blood samples were drawn before and 24 h after intervention. Targeted metabolomics analysis was performed using the AbsoluteIDQ p180 Kit, and changes in metabolite concentrations were compared between the groups. The RIPC group demonstrated significantly different dynamics in nine metabolites compared to the sham group, which generally showed a decrease in metabolite concentrations. The impacted metabolites included glutamate, taurine, the arginine-dimethyl-amide-to-arginine ratio, lysoPC a C24:0, lysoPC a C28:0, lysoPC a C26:1, PC aa C38:1, PC ae C30:2, and PC ae C44:3. RIPC exhibited a ‘stabilization’ effect, maintaining metabolite levels amidst ischemia-reperfusion injuries, suggesting its role in enhancing metabolic control. This may improve outcomes for LEAD patients. However, additional studies are needed to definitively establish causal relationships among these metabolic changes.

## 1. Introduction

Remote ischemic preconditioning (RIPC) is a non-invasive method that aims to protect distant tissues and organs from ischemic injury by harnessing the body’s innate defense mechanisms. Its non-invasive nature, cost-effectiveness, and ease of application make it an attractive option for clinicians to protect organs in various clinical settings against ischemia-reperfusion injury. Performed most often one to four times prior to the ischemia-inducing event, RIPC is administered by inflating and deflating the blood pressure cuff to induce cycles of brief ischemia and reperfusion in a remote tissue, such as the limb. Although the protective effects of ischemic preconditioning were first discovered in 1986, the underlying mechanisms have remained not fully understood to this day [1]. It is believed to involve the release of humoral factors, activation of neural pathways, and systemic anti-inflammatory and antioxidant effects (e.g., expression of antioxidative enzymes) [2,3,4]. These mechanisms collectively grant protection against ischemic injury in remote organs and tissues, including the heart and the kidneys [5,6]. It has been shown that RIPC acts in two stages to protect endothelial cells against ischemia damage in arterial vessels, with an initial phase engaged immediately and lasting up to four hours and an additional phase beginning 24 h after RIPC stimulation and lasting for at least 48 h [2]. While the impact of RIPC has been repeatedly demonstrated in various organs and tissues in animal models, the latest meta-analyses of patients undergoing vascular and endovascular surgical procedures have not provided statistically significant results in clinical trials [7,8]. This controversy is most probably caused by variability in individual responses and heterogeneity of confounding factors in clinical studies. This indicates, firstly, the need for examination of individual differences and, secondly, the necessity to investigate new aspects of the effect of RIPC, including its metabolic profile.

Metabolomics has emerged as an invaluable tool for examining metabolic pathways in response to interventions and for evaluating residual risks across diverse clinical settings, with the goal to improve our understanding of pathophysiology and to potentially facilitate the development of novel therapeutic strategies [9]. In the context of lower extremity arterial disease (LEAD), chronic inflammation is central to its pathogenesis and might be exacerbated by revascularization due to increased production of reactive oxygen species, according to the ischemia-reperfusion injury theory [10]. Research has suggested that RIPC may have the potential to significantly mitigate inflammation related risks associated with interventions, thereby potentially enhancing clinical outcomes for LEAD patients [11]. In our previous research, we demonstrated that patients receiving RIPC prior to vascular surgery exhibited altered acylcarnitine levels, suggesting potential protective effects on mitochondrial function in LEAD patients [12]. Considering that percutaneous transluminal angioplasty (PTA) and digital subtraction angiography (DSA) are likely to introduce fewer confounders than open surgery in similar settings, we hypothesized that the metabolic changes induced by RIPC might be more pronounced in this context.

The aim of this study was to investigate the potential metabolic alterations induced by RIPC in patients undergoing lower limb DSA and/or PTA. We sought to identify specific metabolites and pathways that may be associated with the effects of this intervention, thereby advancing the understanding of the mechanisms by which RIPC might improve outcomes for LEAD patients.

## 2. Materials and Methods

### 2.1. Trial Design

Patients with previously diagnosed LEAD scheduled either for lower limb DSA or PTA or both were included in a double-blinded single-center randomized controlled trial in a non-successive manner. The primary outcome, reported elsewhere [13], was to evaluate the effect of RIPC and sham procedure on arterial stiffness and hemodynamic parameters. Here we report the results of the predetermined secondary outcomes of this study. The trial was approved by the Research Ethics Committee of the University of Tartu and was registered at the U.S. National Institute of Health and the U.S. National Library of Medicine clinical trials register ClinicalTrials.gov (accessed on 16 July 2023) (identifier: NCT02700958). 

### 2.2. Participants

The inclusion and exclusion criteria are listed in Table 1. All participants were recruited between February 2016 and March 2018 from the Department of Vascular Surgery, Tartu University Hospital, Estonia. Written informed consent was provided in the participants’ native language. 

### 2.3. Randomization

The stratified permuted-block randomization technique was used to form six strata combining age (≥75 or <75 years) and the latest available estimated glomerular filtration rate (≥90, 60–89 or 30–59 mL/min/1.73 m^2^). Block size was set to randomly alternate between 2 and 4. Randomization sequence was generated prior to the beginning of the study using the WINPEPI computer program (Version 11, Abramson J.H., Jerusalem, Israel). Results were manually sealed by a third party in opaque envelopes marked by the number of strata and order in the generated sequence. The envelope corresponding to an intervention was opened just before its beginning. 

### 2.4. Interventions

Interventions were made using a standard calibrated blood pressure cuff on the patient’s upper arm. The cuff was inflated to 200 mmHg in the RIPC intervention group and to 20 mmHg in the sham intervention group and was held for 5 min. In both groups inflation of the cuff was repeated for a total of four times with 5 min of perfusion between each cycle. If the patient’s systolic blood pressure was higher than 180 mmHg before the intervention, the blood pressure cuff was filled to 20 mmHg above systolic pressure. Interventions were applied as close as possible to the subsequent endovascular procedure.

### 2.5. Blinding

The cuff’s pressure gauge was concealed in all cases. The patients allocated to the study and the personnel in charge of treatment were blinded to the intervention applied. The statistician developed statistical models and performed the analysis of the groups without the knowledge of the intervention allocated to a group.

### 2.6. Outcomes and Data Cleaning

Blood samples were collected in the morning before the intervention, and as close to 24 h after DSA and/or PTA, on condition that the patient had been fasting for at least 3 h. Blood samples were centrifuged, and serum was stored at −80 °C.

Quantitative and targeted metabolomics assays were performed using the AbsoluteIDQ p180 kit (Biocrates Life Sciences AG, Innsbruck, Austria). The analytical procedure was carried out according to the manufacturer’s standard protocol at the laboratory of the Department of Biochemistry, University of Tartu. Measurements were made using a QTRAP 4500 (ABSciex, Framingham, MA, USA), linked to an Agilent 1260 series HPLC (Agilent Technologies, Santa Clara, CA, USA) with a C18 column, and flow injection analysis. The kit has been previously validated on many instrumental setups [14,15,16]; detailed methodologies are available for all validated instruments from the producer. The range, detection, and quantitation limits for the measured metabolites can be found in Table S1 in the Supplementary Materials. It should be noted that the lipids are annotated at species level, which summarizes the lengths of fatty acid residues and double bonds without providing more precise structural details. For a more comprehensive understanding, readers are encouraged to refer to the latest lipid classification guidelines [17].

To exclude metabolites with too many missing concentration values or values lower than the lower limit of detection, metabolites with less than 67 percent of valid results were removed from analysis. 

### 2.7. Statistical Analysis

Imputation of missing metabolites was performed after data cleaning with the random forest method using the missForest package [18] in the R version 4.2.2 (R Core Team, Vienna, Austria). The RIPC and sham groups were compared using the Mann–Whitney U test; Student´s *t*-test, Wilcoxon rank-sum and Chi-square tests were employed where appropriate using the SPSS version 25 (IBM Corporation, Armonk, NY, USA).

## 3. Results

**Figure 1:**
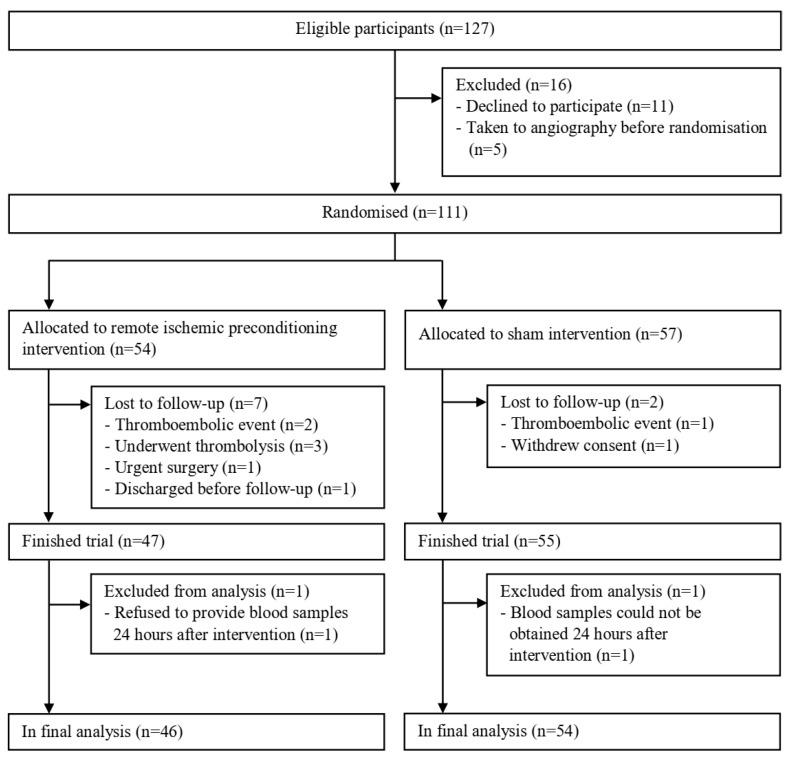
Flow diagram of patient enrolment.

In total, 100 patients were included in final analysis: 46 patients from the RIPC group and 54 patients from the sham group (Figure 1).

The median time from the beginning of intervention to the beginning of DSA and/or PTA was 80 min (IQR 60–118) in the RIPC group and 79 min (IQR 64–112) in the sham group (*p* = 0.377). There was no significant difference between the RIPC and the sham intervention regarding the time spent for DSA and/or PTA (*p* = 0.108), or the time from the beginning of the intervention to the time blood was collected (23 h 49 min and 24 h 13 min; *p* = 0.178, respectively). There was no significant difference in baseline characteristics between the RIPC and sham groups (Table 2). After data cleaning, a total of 150 metabolites and metabolism indicators remained in final analysis (Table S2). Although the baseline measurements revealed significant differences in the levels of glutamate, kynurenine-to-tryptophan ratio, and PC ae C30:2; these differences became non-significant after adjusting for multiple testing.

**Figure 2:**
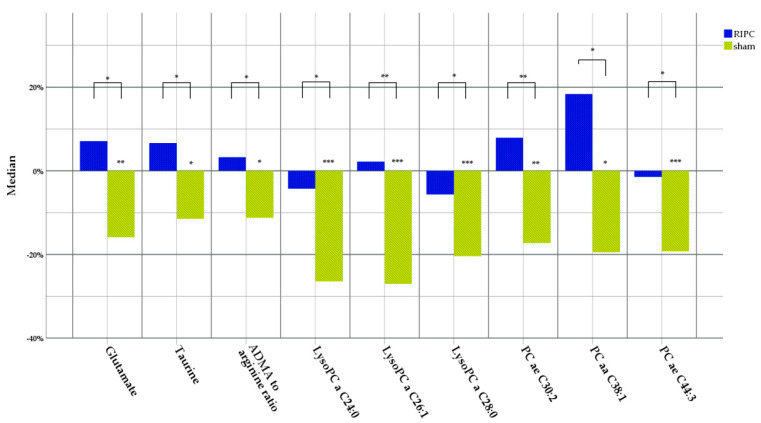
Changes in metabolite levels 24 h post-intervention in the RIPC and sham groups. Changes in metabolite concentrations were calculated by subtracting the baseline measurement values from the 24 h measurement values, and are represented as percentages from baseline measurement. All statistical analyses reflecting changes between the groups were conducted using the Mann–Whitney U test. Only the results that are statistically significant, following the application of the Benja-mini–Hochberg method for multiple comparison correction, are provided. ADMA—asymmetric dimethyl arginine; LysoPC—lysophosphatidylcholine; PC—phosphatidylcholine; a—acyl; aa—diacyl; ae—acyl-alkyl. * *p* < 0.05; ** *p* ≤ 0.01; *** *p* ≤ 0.001. Detailed original concentration values and *p*-values are provided in Table S3 of the Supplementary Materials.

To assess the effect of intervention, we calculated changes in metabolite concentrations and conducted non-parametric tests to ensure the validity of the analysis in the presence of multiple outliers and deviations from normality, as these are better handled by non-parametric methods. Significant changes in the concentrations of several metabolites, including glutamate, taurine, arginine-dimethylamide-to-arginine ratio, lysoPC a C24:0, lysoPC a C28:0, lysoPC a C26:1, PC aa C38:1, PC ae C30:2, and PC ae C44:3 were found between the groups (Figure 2).

To investigate the possible underlying reasons for the observed changes between the groups, we analyzed further changes in metabolite levels within each intervention group. Interestingly, we found decreases in metabolite concentrations in the sham group for all of the above-mentioned metabolites, whereas in the RIPC group no significant changes were detected (Table 3).

## 4. Discussion

Our study explores the metabolic impacts of remote ischemic preconditioning (RIPC) in patients undergoing lower extremity DSA and DSA-PTA. The most noteworthy observation is a ‘stabilization’ effect in the RIPC group, where RIPC was able to maintain levels of various metabolites despite ischemia-reperfusion injuries. This suggests that RIPC could enhance metabolic control, potentially leading to improved clinical outcomes in patients with LEAD.

### 4.1. Taurine

Endogenous taurine is primarily synthesized in the liver and its biosynthesis varies between individuals in relation to the nutritional state, amount of protein intake, and availability of cysteine as the substrate [19]. Taurine inhibits atherogenesis by lowering cholesterol levels and protects endothelial cells from oxidative stress [20]. It stabilizes the membrane´s potential through interference with Na^+^K^+^ATPase, and counteracts ischemic oxidative damage by reducing intracellular calcium levels [20]. Furthermore, taurine deficiency has been associated with muscle atrophy, renal dysfunction, myocardial failure and cardiomyopathy [19,20]. Changes in taurine level between the groups in our study were primarily due to its decreased levels in the sham group, while the levels in the RIPC group remained stable. The decrease in taurine levels after DSA and DSA-PTA might have been caused by several factors in the sham group, such as reduced biosynthesis in the body, genetic factors affecting taurine synthesis, or transportation [19].

Taurine plays a crucial role in various biological functions, including membrane stabilization, osmoregulation, calcium homeostasis, and antioxidant activity; it has been shown to improve vascular function and arterial stiffness across various populations [9,19]. Although there was no significant correlation between taurine and arterial stiffness profile (Table S4), we have shown previously that RIPC improves the hemodynamic profile [13]. The observed ‘stabilization’ of taurine levels implies a potential role for RIPC in preserving taurine concentrations, which could help alleviate the health concerns associated with taurine deficiency. This hints at a possible function of taurine in mediating the protective effects of RIPC in LEAD patients.

### 4.2. Asymmetric Dimethyl Arginine and Arginine

Although there occurred no significant changes in asymmetric dimethyl arginine (ADMA) or L-arginine (Arg) levels between the study groups, there was difference in the ADMA/Arg ratio. Total dimethyl arginine (DMA), ADMA, citrulline/Arg ratio and citrulline levels decreased only in the sham group. These findings indicate that the likely cause of the observed changes in the ADMA/Arg ratio between the study groups was mainly due to the decrease in ADMA levels in the sham group. The decrease in ADMA levels in the sham group could be attributed to changes in ADMA synthesis and removal processes, involving factors such as enzymatic activity of dimethylaminohydrolase and changes in renal function. However, without direct measurements of these enzymatic activities, the exact mechanisms underlying these observed changes remain speculative.

Previous studies have suggested that RIPC might modify the citrulline–nitric oxide cycle by increasing the activity of argininosuccinate synthetase 1 and decreasing arginase activity, which could lead to an increased Arg/Orn ratio and elevated Arg levels that can be directly used for nitric oxide (NO) production [21]. However, since ADMA, a well-known nitric oxide synthase (NOS) inhibitor, decreased only in the sham group, the RIPC group might have relatively low NOS activity and NO production compared to the sham group. The decrease in circulating citrulline levels further emphasizes this, as citrulline is converted to arginine in proximal kidney tubule cells [22], indicating improved citrulline delivery to the kidney and arginine turnover in patients of the sham group. The unaffected ADMA/Arg ratio could suggest that RIPC plays a role in maintaining this ratio, potentially affecting NO production and could contribute to a healthy endothelial function.

Previous metabolomics research has shown that deficiency of tetrahydrobiopterin (BH4), an essential cofactor of NOS, is common in symptomatic LEAD patients due to its depletion caused by oxidative stress and inflammation [23]. These reduced levels of BH4 can impair NO production, leading to endothelial dysfunction [24]. BH4 is also a cofactor for phenylalanine hydroxylase, which converts phenylalanine to tyrosine [23]. A decreased tyrosine-to-phenylalanine ratio has been associated with oxidative stress, inflammation, and a decreased ankle brachial index [23]. In our study, both groups exhibited a similar increase in the tyrosine-to-phenylalanine ratio. This suggests greater availability of BH4 not only for the phenylalanine hydroxylase but also for the NOS. Furthermore, the changes observed might suggest a decrease in oxidative stress in general, as well as an enhancement in blood supply following the DSA and DSA-PTA procedures.

### 4.3. Glutamate

Data on metabolic changes in human skeletal muscles after ischemia-reperfusion injury are scarce. Glutamate serves as an intersection between carbohydrate and amino acid metabolism, while acting also as a source for all precursors of intestinal citrulline synthesis [22]. A significant decrease in glutamate levels during maximal ischemia and 24 h hours after lower limb surgery (−29% and −51%, respectively) has been described earlier, indicating that glutamate usage is upregulated both during and after ischemia [25]. Elevated glutamate levels following RIPC have been previously observed in both human and animal studies, implying an enhancement in energy metabolism to accommodate increased demand [21,26,27]. Our results align with these findings, as a significant change in glutamate levels was noted between the groups, with levels only reduced in the sham group. A reduction in glutamate levels could indicate its uptake by cells, as glutamate can be readily utilized through alpha-ketoglutarate in the Krebs cycle as an energy source. Stabilization of glutamate levels in the context of RIPC suggests an ability to regulate and balance the metabolic demand, potentially preventing excessive utilization of glutamate as an energy source. The observed changes underline the complex interactions of metabolic pathways and enzymes in amino acids, providing further insights into the mechanisms through which RIPC operates.

### 4.4. Lysophosphatidylcholines and Phosphatidylcholines

LysoPCs and PCs are the phospholipids that serve as essential components of cell membranes and play crucial roles in various cellular functions [28]. PCs modulate membrane fluidity and participate in cell signalling; they also are important components of the lung surfactant and regulate lipid homeostasis in the liver. PCs are primarily synthesized in the endoplasmic reticulum through two main pathways: the cytidine 5-diphosphocholine (CDP-choline) pathway and the phosphatidylethanolamine methyltransferase (PEMT) pathway.

Phosphatidylcholine (PC) is composed of a glycerol backbone, two fatty acid chains, a phosphate group, and a choline molecule. A fatty acyl residue, connected by an ester bond, is represented by an “a” in the name of PC. If the fatty alcohol residue in the sn-1 position is present instead of a fatty acyl residue, the name of PC includes an “e”. The first number in the names of PC and lysoPC indicates the total number of carbon atoms, while the second number represents the number of double bonds.

PCs undergo hydrolysis by phospholipase A2 (PLA2), resulting in the formation of lysoPCs and fatty acids [29]. LysoPCs are also formed by lecithin cholesterol acyltransferase (LCAT) activity in high density lipoproteins (HDL) and oxidation of low density lipoproteins (LDL) [30]. Unlike PC, lysoPC contains a glycerol backbone with only one fatty acid chain, a phosphate group, and a choline molecule. LysoPCs play a role in various physiological processes, including cell signalling, inflammation, and immune responses.

The role of lysoPC in clinical settings is conflicting, as both its pro- and anti-inflammatory properties have been reported previously [9,31,32]. In our study significant changes were only seen in long and very long chain lysoPCs (C24:0, C26:1 and C28:0) and PCs (C30:2, C38:1 and C44:3) between the groups. Notably, the significant changes in lysoPCs and PCs did not involve highly unsaturated forms, likely due to their use for beta-oxidation necessitating more complex pathways. As the changes occurred mainly in the sham group, this could reflect an increased rate of catabolism and a more efficient removal of lysoPCs from the bloodstream and into a variety of tissues [33]. This suggests more balanced energy usage as a consequence of the RIPC procedure. However, as the circulating levels of lysoPCs are determined by a combination of their production, clearance, and degradation, we could not establish the direct cause of the changes found in this study, since specific enzyme activities were not measured.

We found that lysoPC and PC levels remained stable in the RIPC group, suggesting RIPC’s role in preserving membrane integrity and function. Contrarily, similar patients undergoing vascular surgery did not exhibit significant changes in lysoPCs and PCs. This may be due to the greater metabolic disturbance from open surgery compared to the relatively less invasive stent placement procedure [13,26,34]. As the research of lysoPCs used as signal molecules is still scarce, further studies are needed to fully comprehend the changes and pathways seen in the RIPC group.

### 4.5. Adiponectin and Its Effect on the Metabolomical Profile

We have previously shown that RIPC significantly limited the increase in adiponectin levels in the same patient group [34]. Adiponectin has been shown to mediate insulin sensitizing effects mainly by suppressing gluconeogenesis in the liver and by stimulating fatty acid oxidation [35]. In addition, through suppressing the endoplasmic reticulum, adiponectin has been shown to induce autophagy in skeletal muscle cells [36]. Although autophagy is essential for maintaining cellular homeostasis, excessive and dysregulated autophagy in acute situations may contribute to further muscle deterioration, as it is upregulated already within two hours after ischemia [37,38]. As noted above, several changes in the metabolomical profile of the sham group can indicate an increased uptake and utilization of metabolites, possibly to meet the increased energy demand in catabolic settings. However, the group of remote ischemic preconditioning remains relatively stable. Similar results have been obtained previously on rodents where ischemic preconditioning attenuated brain ischemia-reperfusion damage by limiting hyperglycolysis and affecting the expression of genes associated with energy metabolism [39,40]. Improvement in energy usage following RIPC has also been shown in humans, indicating preserved mitochondrial function after injurious event and suggesting improved tolerance of ischemia-reperfusion injury [41]. The unchanged adiponectin levels in the RIPC group might imply that RIPC prevents the excessive upregulation of autophagy and catabolic activity, thereby contributing to the maintenance of metabolic balance. As a result, RIPC may potentially mitigate the adverse effects associated with medical interventions and improve the metabolic profile of LEAD patients.

### 4.6. Limitations

Despite the intriguing findings, there are several limitations to our study that should be considered. First, sample size was relatively small, which may have limited the statistical power to detect subtler differences between the study groups. In addition, even though all patients were randomized the potential influence of confounding factors, such as comorbidities and concomitant medications, cannot be completely ruled out as they might have influenced the observed changes. Lastly, the observed metabolic changes may not directly translate to clinical benefits. Further research is needed to establish a clear link between the metabolic alterations induced by RIPC and the improved clinical outcomes in LEAD patients.

## 5. Conclusions

In conclusion, our study sheds light on the metabolic alterations triggered by RIPC in patients undergoing lower limb DSA and DSA-PTA, emphasizing the stabilizing effects of RIPC on various metabolites. This stabilization may enhance metabolic control, potentially leading to improved clinical outcomes for LEAD patients. These findings provide novel insights into RIPC’s metabolic impacts and pave the way for the development of personalized approaches to mitigate the metabolic disruptions in LEAD patients. Further studies are required to fully elucidate the mechanisms through which RIPC mediates these effects, which will be crucial for harnessing the full potential of RIPC in clinical settings.

## Figures and Tables

**Table 1:** Inclusion and exclusion criteria.

Inclusion criteria
Previously diagnosed LEAD hospitalized for DSA and/or PTA Written informed consent
Exclusion criteria
Age < 18 years Latest available eGFR < 30 mL/min/1.73 m^2^ Simultaneous participation in any other clinical trial Coexisting pathology of the upper limbs limiting the use of the blood pressure measuring cuff Active malignant tumor (in remission < 5 years or ongoing treatment) Documented allergic reaction to iodinated contrast agent Acute infection (body temperature ≥ 38 °C, CRP ≥ 50 mg/L) Cardiac rhythm abnormalities (atrial fibrillation and flutter, frequent supraventricular and ventricular complexes) Home-based oxygen treatment Inability to lie supine < 40 min History of vascular surgery in the axillary region Documented upper limb deep vein thrombosis

LEAD—lower extremity arterial disease, DSA—digital subtraction angiography, PTA—percutaneous transluminal angioplasty, eGFR—estimated glomerular filtration rate, CRP—C-reactive protein.

**Table 2:** Baseline characteristics of the study population.

Characteristics	RIPC (n = 46)		SHAM (n = 54)		*p*-Value
	Mean/ Median	SD/IQR	Mean/ Median	SD/IQR	
**Demographic**					
** Male (n)**	33 (71.7%)		47 (87.0%)		0.098
** Mean age (y)**	66.1	±10.3	65.0	±11.4	0.61
** Weight (kg)**	75.2	±17.3	78.0	±16.7	0.42
** Body mass index (kg/m^2^)**	25.4	(22.7–30.0)	25.3	(23.5–29.4)	0.66
**Renal function at inclusion**					
** eGFR <90 (n) ^#^**	27 (58.7%)		30 (55.6%)		0.91
** 60–89 (n) ^#^**	19 (41.3%)		20 (37.0%)		
** 30–59 (n) ^#^**	8 (18.5%)		10 (18.5%)		
**History of smoking (n) ^†^**	35 (76.1%)		41 (75.9%)		1
**Concomitant diseases**					
** Stage of LEAD III or more ^‡^**	23 (50.0%)		25 (46.3%)		0.87
** Stage of LEAD III (n) ^‡^**	9 (19.6%)		10 (18.5%)		
** Stage of LEAD IV (n) ^‡^**	14 (30.4%)		15 (27.8%)		
** Diabetes (n)**	10 (21.7%)		13 (24.1%)		0.97
** Hypertension (n) ^◊^**	32 (69.6%)		28 (51.9%)		0.11
**Medications**					
** ACE inhibitors (n)**	18 (39.1%)		14 (25.9%)		0.16
** ARBs (n)**	12 (26.1%)		11 (20.4%)		0.50
** Calcium channel blockers (n)**	18 (39.1%)		15 (29.8%)		0.23
** Beta blockers (n)**	12 (26.1%)		12 (22.2%)		0.65
** Diuretics (n)**	16 (34.8%)		12 (22.2%)		0.16
** Antiagregants (n)**	24 (52.2%)		26 (48.1%)		0.69
** Anticoagulants (n)**	1 (2.2%)		1 (1.9%)		0.91
** Naftidrofuryl/pentoxifylline (n)**	33 (71.7%)		35 (64.8%)		0.46
** Statins (n)**	18 (39.1%)		16 (29.6%)		0.39
** Insulin therapy (n)**	6 (13.0%)		8 (14.8%)		0.80
** Oral antidiabetic agents (n)**	3 (6.5%)		5 (9.3%)		0.62
**Creatinine (μmol/L)**	78	(65–92)	77	(67–92)	0.80
**eGFR (mL/min/1.73 m^2^)**	84	(68–94)	91	(69–100)	0.17
**Urea (mmol/L)**	5.0	(4.4–6.6)	5.5	(4.4–6.6)	0.76
**Cholesterol (mmol/L)**	4.66	±1.38	4.85	±1.42	0.52
**HDL (mmol/L)**	1.17	(0.96–1.55)	1.12	(0.94–1.45)	0.58
**LDL (mmol/L)**	2.70	(2.07–3.63)	3.02	(2.05–3.91)	0.50
**TG (mmol/L)**	1.30	(0.98–2.06)	1.43	(1.1–1.98)	0.36
**Adiponectin (ng/mL)**	6322	(3769–8523)	5541	(3327–9406)	0.48

†—current and ex-smokers; ‡—Stage of LEAD by Fontaine’s classification; ^◊^—on medication; ^#^—ml/min/1.73 m^2^; y—years of age; LEAD—lower extremity arterial disease; eGFR—estimated glomerular filtration rate; ACE—angiotensin-converting enzyme; ARB—angiotensin receptor blocker; HDL—high-density lipoprotein; LDL—low-density lipoprotein; TG—triglycerides.

**Table 3:** Significant changes in metabolite levels 24 h after intervention within the study groups.

	Group	Change	IQR/SD	*p*-Value
**Asymmetric dimethyl arginine**	SHAM	−0.075	(−0.367–0.217)	0.005
**Citrulline-to-arginine ratio**	SHAM	−0.042	(−0.157–0.074)	0.003
**Citrulline**	SHAM	−5.3	(−25.2–14.6)	0.003
**Glutamate**	SHAM	−18.0	(−66.3–30.3)	0.004
**Total dimethylamide**	SHAM	−0.173	(−0.876–0.530)	0.001
**Tyrosine**	RIPC	−9.8	(−32.5–12.9)	0.004
**Tyrosine-to-phenylalanine ratio** ^♦^	SHAM	0.08	0.20	0.004
**Tyrosine-to-phenylalanine ratio** ^♦^	RIPC	0.12	0.19	<0.001
**LysoPC a C16:0**	SHAM	−25.0	(−89.6–39.7)	0.003
**LysoPC a C17:0**	SHAM	−0.59	(−1.91–0.73)	<0.001
**LysoPC a C18:0**	SHAM	−4.9	(−15.3–5.6)	<0.001
**LysoPC a C18:0**	RIPC	−6.2	(−20.2–7.8)	0.002
**LysoPC a C18:1**	SHAM	−8.8	(−23.3–5.7)	<0.001
**LysoPC a C18:1**	RIPC	−7.07	(−26.1–11.9)	0.001
**LysoPC a C18:2**	SHAM	−16.1	(−48.4–16.2)	<0.001
**LysoPC a C18:2**	RIPC	−13.0	(−50.4–24.3)	<0.001
**LysoPC a C20:3**	SHAM	−0.94	(−2.74–0.87)	<0.001
**LysoPC a C20:3**	RIPC	−1.0	(−3.5–1.4)	0.001
**LysoPC a C20:4**	SHAM	−2.5	(−7.5–2.5)	<0.001
**LysoPC a C20:4**	RIPC	−3.3	(−11.3–4.7)	0.002
**LysoPC a C24:0**	SHAM	−0.21	(−0.73–0.32)	0.001
**LysoPC a C26:1**	SHAM	−0.25	(−1.04–0.55)	0.001
**LysoPC a C28:0**	SHAM	−0.23	(−0.86–0.39)	0.001
**PC ae C30:0**	SHAM	−0.026	(−0.132–0.081)	0.005
**PC ae C30:2**	SHAM	−0.027	(−0.123–0.069)	0.002
**PC ae C34:3**	RIPC	−0.75	(−2.30–0.80)	0.001
**PC ae C42:1**	SHAM	−0.094	(−0.323–0.136)	0.005
**PC ae C44:3**	SHAM	−0.041	(−0.154–0.071)	0.001

The reported results include only the statistically significant findings that have been adjusted for multiple comparisons using the Benjamini–Hochberg method. The changes in metabolite concentrations were calculated by subtracting the baseline measurement values from the 24 h measurement values and are given in units of micromolar (μM). Median values are shown if not otherwise indicated. ^♦^—mean changes and standard deviations are given. lysoPC- lysophosphatidylcholine; PC—phosphatidylcholine; a—acyl; aa—diacyl; ae, acyl-alkyl, IQR—interquartile range, SD—standard deviation.

## Data Availability

The data presented in this study are available on request from the corresponding author. The data are not publicly available due to ethical restrictions.

## Supplementary Materials

The following supporting information can be downloaded at: https://www.mdpi.com/article/10.3390/metabo13070856/s1, Table S1: Range, detection, and quantitation limits for measured metabolites; Table S2: Baseline measurements of metabolites; Table S3: Significant changes in metabolite levels 24 h after intervention between the RIPC and sham group; Table S4: Correlations of significant hemodynamic, biochemical and metabolic changes.
