# Supplementary material for: Effects of RIPC on the Metabolomical Profile during Lower Limb Digital Subtraction Angiography: A Randomized Controlled Trial
Source: Metabolites. 2023 Jul 18;13(7):856. doi: 10.3390/metabo13070856 (PMC10384110; doi:10.3390/metabo13070856)
Supplement: Supplementary file 1 [file metabolites-13-00856-s001.zip › Table S1 - Range, detection, and quantitation limits for measured metabolites.pdf]

**Table S1.** Range, detection, and quantitation limits for measured metabolites.

| | **Lowest Sample** | **Highest Sample** | **Lower Limit of Detection (µM)** | **Lower Limit of Quantification (µM)** |
|---|---|---|---|---|
| **C0** | 24.19 | 234.32 | 10.2 | 10.2 |
| **C14:1** | 0.17 | 2.49 | 0.021 | 0.021 |
| **C18:1** | 0.09 | 0.56 | 0.078 | 0.078 |
| **Ala** | 162.19 | 2182.50 | 1 | 20 |
| **Arg** | 57.46 | 490.81 | 0.5 | 5 |
| **Asn** | 130.26 | 1008.13 | 1.5 | 5 |
| **Asp** | 4.15 | 274.06 | 1.5 | 5 |
| **Cit** | 13.38 | 221.20 | 1 | 5 |
| **Gln** | 295.93 | 4254.90 | 1.5 | 20 |
| **Glu** | 6.50 | 422.65 | 2 | 10 |
| **Gly** | 129.40 | 2291.49 | 0.5 | 25 |
| **His** | 48.69 | 385.54 | 0.5 | 5 |
| **Ile** | 50.21 | 397.68 | 0.5 | 5 |
| **Leu** | 88.48 | 743.53 | 1.5 | 50 |
| **Lys** | 143.45 | 1024.25 | 0.5 | 10 |
| **Met** | 14.58 | 137.47 | 0.1 | 5 |
| **Orn** | 49.99 | 595.25 | 0.5 | 5 |
| **Phe** | 41.06 | 341.23 | 0.1 | 5 |
| **Ser** | 66.05 | 706.22 | 1 | 5 |
| **Thr** | 210.40 | 1865.27 | 0.5 | 5 |
| **Trp** | 21.67 | 267.54 | 0.5 | 5 |
| **Tyr** | 28.00 | 360.42 | 0.5 | 5 |
| **Val** | 126.42 | 1324.78 | 0.5 | 10 |

| | | | | |
|---|---|---|---|---|
| **Ac-Orn** | 0.52 | 15.19 | 0.2 | 1 |
| **ADMA** | 0.20 | 3.25 | 0.08 | 0.25 |
| **Creatinine** | 4.25 | 334.21 | 1 | 10 |
| **Kynurenine** | 1.72 | 21.00 | 0.3 | 1 |
| **Putrescine** | 0.04 | 0.97 | 0.02 | 0.1 |
| **Serotonin** | 0.13 | 4.14 | 0.03 | 0.1 |
| **Taurine** | 43.93 | 602.38 | 0.8 | 2.5 |
| **Total DMA** | 1.06 | 11.89 | 0.1 | 1.25 |
| **LysoPC a C16:0** | 90.92 | 811.22 | 0.105 | 0.105 |
| **LysoPC a C16:1** | 1.94 | 20.16 | 0.059 | 0.059 |
| **LysoPC a C17:0** | 0.99 | 19.11 | 0.02 | 0.02 |
| **LysoPC a C18:0** | 12.48 | 146.48 | 0.255 | 0.255 |
| **LysoPC a C18:1** | 14.15 | 162.37 | 0.035 | 0.035 |
| **LysoPC a C18:2** | 21.84 | 322.05 | 0.105 | 0.105 |
| **LysoPC a C20:3** | 1.50 | 18.67 | 0.026 | 0.026 |
| **LysoPC a C20:4** | 5.64 | 57.16 | 0.02 | 0.02 |
| **LysoPC a C24:0** | 0.25 | 6.72 | 0.05 | 0.05 |
| **LysoPC a C26:0** | 0.29 | 8.56 | 0.07 | 0.07 |
| **LysoPC a C26:1** | 0.22 | 8.07 | 0.018 | 0.018 |
| **LysoPC a C28:0** | 0.17 | 10.62 | 0.127 | 0.127 |
| **LysoPC a C28:1** | 0.28 | 5.45 | 0.009 | 0.009 |
| **PC aa C24:0** | 0.13 | 5.45 | 0.043 | 0.043 |
| **PC aa C26:0** | 0.90 | 22.70 | 0.838 | 0.838 |
| **PC aa C28:1** | 1.41 | 13.31 | 0.081 | 0.081 |
| **PC aa C30:0** | 1.39 | 35.61 | 0.128 | 0.128 |
| **PC aa C30:2** | 0.05 | 2.56 | 0 | 0 |

| | | | | |
|---|---|---|---|---|
| **PC aa C32:0** | 6.25 | 112.57 | 0.02 | 0.02 |
| **PC aa C32:1** | 4.90 | 266.08 | 0.005 | 0.005 |
| **PC aa C32:2** | 0.18 | 25.96 | 0.013 | 0.013 |
| **PC aa C32:3** | 0.18 | 2.83 | 0.008 | 0.008 |
| **PC aa C34:1** | 105.91 | 2299.28 | 0.033 | 0.033 |
| **PC aa C34:2** | 175.79 | 2557.55 | 0.054 | 0.054 |
| **PC aa C34:3** | 4.40 | 138.82 | 0.012 | 0.012 |
| **PC aa C34:4** | 0.48 | 11.81 | 0.014 | 0.014 |
| **PC aa C36:0** | 0.78 | 15.32 | 0.317 | 0.317 |
| **PC aa C36:1** | 13.58 | 441.70 | 0.014 | 0.014 |
| **PC aa C36:2** | 77.26 | 1521.85 | 0.016 | 0.016 |
| **PC aa C36:3** | 40.90 | 761.52 | 0.011 | 0.011 |
| **PC aa C36:4** | 72.84 | 995.09 | 0.01 | 0.01 |
| **PC aa C36:5** | 11.76 | 257.12 | 0.003 | 0.003 |
| **PC aa C36:6** | 0.30 | 7.30 | 0.003 | 0.003 |
| **PC aa C38:0** | 1.02 | 13.75 | 0.005 | 0.005 |
| **PC aa C38:1** | 0.10 | 12.72 | 0.008 | 0.008 |
| **PC aa C38:3** | 13.13 | 267.29 | 0.009 | 0.009 |
| **PC aa C38:4** | 29.80 | 578.75 | 0.01 | 0.01 |
| **PC aa C38:5** | 19.75 | 340.12 | 0.011 | 0.011 |
| **PC aa C38:6** | 30.19 | 504.60 | 0.393 | 0.393 |
| **PC aa C40:2** | 0.07 | 3.06 | 0.005 | 0.005 |
| **PC aa C40:3** | 0.20 | 4.58 | 0.002 | 0.002 |
| **PC aa C40:4** | 0.79 | 23.62 | 0.011 | 0.011 |
| **PC aa C40:5** | 2.52 | 62.68 | 0.002 | 0.002 |
| **PC aa C40:6** | 10.15 | 235.49 | 0.281 | 0.281 |

| | | | | |
|---|---|---|---|---|
| **PC aa C42:0** | 0.19 | 2.24 | 0.031 | 0.031 |
| **PC aa C42:1** | 0.08 | 1.59 | 0.007 | 0.007 |
| **PC aa C42:2** | 0.09 | 1.80 | 0.056 | 0.056 |
| **PC aa C42:4** | 0.05 | 0.88 | 0.006 | 0.006 |
| **PC aa C42:5** | 0.07 | 2.76 | 0.001 | 0.001 |
| **PC aa C42:6** | 0.16 | 3.19 | 0.278 | 0.278 |
| **PC ae C30:0** | 0.16 | 3.54 | 0.134 | 0.134 |
| **PC ae C30:1** | 0.08 | 4.81 | 0.018 | 0.018 |
| **PC ae C30:2** | 0.05 | 1.26 | 0.006 | 0.006 |
| **PC ae C32:1** | 1.15 | 22.93 | 0.005 | 0.005 |
| **PC ae C32:2** | 0.32 | 4.55 | 0.016 | 0.016 |
| **PC ae C34:0** | 0.43 | 13.58 | 0.025 | 0.025 |
| **PC ae C34:1** | 3.68 | 84.57 | 0.014 | 0.014 |
| **PC ae C34:2** | 3.77 | 64.81 | 0.008 | 0.008 |
| **PC ae C34:3** | 2.11 | 33.49 | 0.027 | 0.027 |
| **PC ae C36:0** | 0.35 | 5.96 | 0.139 | 0.139 |
| **PC ae C36:1** | 2.95 | 58.63 | 0.121 | 0.121 |
| **PC ae C36:2** | 4.79 | 93.16 | 0.028 | 0.028 |
| **PC ae C36:3** | 2.45 | 46.64 | 0.004 | 0.004 |
| **PC ae C36:4** | 8.33 | 102.99 | 0.016 | 0.016 |
| **PC ae C36:5** | 4.32 | 49.25 | 0.005 | 0.005 |
| **PC ae C38:0** | 0.50 | 9.80 | 0.103 | 0.103 |
| **PC ae C38:1** | 0.03 | 4.03 | 0.014 | 0.014 |
| **PC ae C38:2** | 0.58 | 16.63 | 0.006 | 0.006 |
| **PC ae C38:3** | 1.35 | 23.19 | 0.012 | 0.012 |
| **PC ae C38:4** | 5.58 | 81.41 | 0.016 | 0.016 |

| | | | | |
|---|---|---|---|---|
| **PC ae C38:5** | 6.70 | 85.31 | 0.023 | 0.023 |
| **PC ae C38:6** | 2.80 | 38.27 | 0.001 | 0.001 |
| **PC ae C40:1** | 0.42 | 9.57 | 0.008 | 0.008 |
| **PC ae C40:2** | 0.62 | 7.93 | 0.01 | 0.01 |
| **PC ae C40:3** | 0.30 | 4.53 | 0.008 | 0.008 |
| **PC ae C40:4** | 0.72 | 12.88 | 0.049 | 0.049 |
| **PC ae C40:5** | 1.01 | 16.55 | 0.003 | 0.003 |
| **PC ae C40:6** | 1.55 | 28.21 | 0.013 | 0.013 |
| **PC ae C42:1** | 0.16 | 3.67 | 0.094 | 0.094 |
| **PC ae C42:2** | 0.20 | 3.54 | 0.006 | 0.006 |
| **PC ae C42:3** | 0.28 | 5.10 | 0.011 | 0.011 |
| **PC ae C42:4** | 0.18 | 3.57 | 0.3 | 0.3 |
| **PC ae C42:5** | 0.62 | 8.73 | 0.623 | 0.623 |
| **PC ae C44:3** | 0.06 | 1.48 | 0.025 | 0.025 |
| **PC ae C44:4** | 0.08 | 1.78 | 0.075 | 0.075 |
| **PC ae C44:5** | 0.50 | 6.91 | 0.065 | 0.065 |
| **PC ae C44:6** | 0.34 | 5.07 | 0.035 | 0.035 |
| **SM (OH) C14:1** | 1.90 | 20.95 | 0.012 | 0.012 |
| **SM (OH) C16:1** | 0.89 | 11.60 | 0.008 | 0.008 |
| **SM (OH) C22:1** | 3.68 | 35.91 | 0.004 | 0.004 |
| **SM (OH) C22:2** | 2.88 | 28.31 | 0.01 | 0.01 |
| **SM (OH) C24:1** | 0.30 | 3.48 | 0.002 | 0.002 |
| **SM C16:0** | 37.68 | 437.68 | 0.03 | 0.03 |
| **SM C16:1** | 4.33 | 52.46 | 0.015 | 0.015 |
| **SM C18:0** | 7.74 | 90.27 | 0.008 | 0.008 |
| **SM C18:1** | 2.57 | 31.21 | 0.01 | 0.01 |

| | | | | |
|---|---|---|---|---|
| **SM C20:2** | 0.07 | 0.71 | 0.003 | 0.003 |
| **SM C24:0** | 4.38 | 61.68 | 0.003 | 0.003 |
| **SM C24:1** | 13.43 | 182.41 | 0.001 | 0.001 |
| **SM C26:0** | 0.02 | 0.83 | 0.004 | 0.004 |
| **SM C26:1** | 0.08 | 2.57 | 0.004 | 0.004 |
| **H1** | 3623.85 | 26918.34 | 29.9 | 200 |
