# Supplementary material for: Effects of RIPC on the Metabolomical Profile during Lower Limb Digital Subtraction Angiography: A Randomized Controlled Trial
Source: Metabolites. 2023 Jul 18;13(7):856. doi: 10.3390/metabo13070856 (PMC10384110; doi:10.3390/metabo13070856)
Supplement: Supplementary file 1 [file metabolites-13-00856-s001.zip › Table S2 - Baseline measurements of metabolites.pdf]

Table S2. Baseline measurements of metabolites.

	RIPC		SHAM		
	**Mean/Median**	**±SD/IQR**	**Mean/Median**	**±SD/IQR**	***p*-Value**
**C0**	51.70	18.19	47.87	19.08	.19
**C14:1**	0.47	0.44	0.47	0.25	.86
**C18:1**	0.18	0.08	0.19	0.06	.80
**Ala**	456.00	198.02	509.65	168.02	.38
**Arg**	140.00	47.02	136.26	50.07	.84
**Asn**	312.95	±95.57	305.48	120.88	.67
**Asp**	73.20	35.62	57.04	51.29	.13
**Cit**	49.10	21.68	45.91	928.64	.67
**Gln**	945.00	343.86	928.64	295.53	.72
**Glu**	94.25	73.51	117.33	60.02	.018
**Gly**	372.50	128.94	358.34	141.42	.28
**His**	105.50	30.77	103.44	32.93	.94
**Ile**	102.05	29.27	102.52	38.08	.55
**Leu**	205.00	90.41	210.13	82.86	.35
**Lys**	300.00	132.33	308.49	116.97	.53
**Met**	33.50	11.86	34.66	15.47	.60
**Orn**	124.00	37.29	124.87	43.06	.68
**Phe**	83.20	19.64	82.00	24.74	.54
**Ser**	175.96	±48.44	162.32	53.16	.54
**Thr**	599.00	269.49	575.96	246.64	.98
**Trp**	71.70	28.74	75.41	26.42	.22
**Tyr**	89.65	46.17	95.00	34.64	.75
**Val**	345.49	±108.05	350.78	153.94	.24
**Ac-Orn**	1.91	1.03	1.92	0.79	.42
**ADMA**	0.71	0.17	0.67	0.25	.51
**Creatinine**	122.00	25.66	122.00	16.11	.88
**Kynurenine**	4.16	2.13	3.97	1.75	.51
**Putrescine**	0.21	0.10	0.22	0.12	.35
**Serotonin**	0.75	0.62	0.81	0.51	.56
**Taurine**	117.50	46.96	118.88	43.05	.68
**Total DMA**	2.46	0.83	2.24	0.82	.33
**LysoPC a C16:0**	241.00	117.45	251.54	111.43	.53
**LysoPC a C16:1**	5.67	3.39	5.42	2.89	.28
**LysoPC a C17:0**	4.19	1.93	3.95	2.00	.97
**LysoPC a C18:0**	37.80	24.40	39.19	22.50	.89
**LysoPC a C18:1**	47.50	27.42	46.94	24.46	.71
**LysoPC a C18:2**	75.20	55.23	76.59	45.35	.85

**LysoPC a C20:3**	5.36	3.63	5.24	2.80	.47
**LysoPC a C20:4**	17.90	7.94	16.98	8.67	.72
**LysoPC a C24:0**	0.72	0.41	0.78	0.54	.24
**LysoPC a C26:0**	1.16	0.69	1.25	1.16	.36
**LysoPC a C26:1**	0.80	0.95	0.99	0.99	.56
**LysoPC a C28:0**	0.82	0.57	0.89	0.80	.20
**LysoPC a C28:1**	0.89	0.49	0.96	0.74	.65
**PC aa C24:0**	0.47	0.57	0.51	0.52	.20
**PC aa C26:0**	2.50	1.42	2.77	1.92	.29
**PC aa C28:1**	3.02	1.21	3.07	1.37	.39
**PC aa C30:0**	4.65	2.67	5.08	2.97	.52
**PC aa C30:2**	0.62	0.45	0.60	0.42	.85
**PC aa C32:0**	17.15	8.86	18.71	9.74	.69
**PC aa C32:1**	23.20	17.87	24.56	20.60	.95
**PC aa C32:2**	4.77	3.21	4.42	3.74	.61
**PC aa C32:3**	0.52	±0.18	0.52	0.24	.71
**PC aa C34:1**	292.00	136.29	301.11	175.45	.65
**PC aa C34:2**	478.00	±232.15	491.34	218.98	.39
**PC aa C34:3**	17.55	11.32	17.89	9.31	.99
**PC aa C34:4**	1.65	1.15	1.59	0.86	.65
**PC aa C36:0**	2.77	0.87	2.72	2.06	.44
**PC aa C36:1**	54.25	34.38	53.18	30.97	.87
**PC aa C36:2**	254.00	139.06	256.83	127.66	.86
**PC aa C36:3**	129.00	66.59	132.71	60.38	.86
**PC aa C36:4**	208.50	118.07	207.58	107.79	.81
**PC aa C36:5**	37.30	26.30	47.31	36.99	.25
**PC aa C36:6**	1.50	0.70	1.58	0.97	.87
**PC aa C38:0**	2.79	1.22	2.70	1.59	.97
**PC aa C38:1**	1.03	0.71	1.12	0.93	.15
**PC aa C38:3**	48.30	31.89	49.70	19.89	.89
**PC aa C38:4**	117.00	71.10	108.05	59.60	.44
**PC aa C38:5**	55.35	32.15	57.20	27.42	.92
**PC aa C38:6**	91.40	46.97	93.20	51.87	.45
**PC aa C40:2**	0.34	0.13	0.38	0.24	.35
**PC aa C40:3**	0.58	0.24	0.58	0.33	.96
**PC aa C40:4**	3.15	1.58	3.01	1.25	.55
**PC aa C40:5**	3.45	3.45	8.73	4.16	.59
**PC aa C40:6**	32.20	20.62	32.69	20.09	.92
**PC aa C42:0**	0.55	0.22	0.49	0.27	.56
**PC aa C42:1**	0.28	0.09	0.26	0.12	.15
**PC aa C42:2**	0.27	0.12	0.30	0.13	.23

**PC aa C42:4**	0.16	0.06	0.15	0.08	.71
**PC aa C42:5**	0.35	0.15	0.36	0.50	.81
**PC aa C42:6**	0.49	0.09	0.52	±0.19	.58
**PC ae C30:0**	0.43	0.17	0.48	0.24	.19
**PC ae C30:1**	0.52	0.30	0.64	0.64	.12
**PC ae C30:2**	0.15	0.09	0.17	0.13	.036
**PC ae C32:1**	3.19	0.93	3.19	1.89	.92
**PC ae C32:2**	0.86	0.33	0.95	0.46	.37
**PC ae C34:0**	1.45	0.61	1.54	0.74	.98
**PC ae C34:1**	10.85	4.85	10.77	5.29	.79
**PC ae C34:2**	10.55	3.69	10.34	5.32	.67
**PC ae C34:3**	6.98	±3.08	6.01	3.20	.39
**PC ae C36:0**	1.08	0.47	1.03	0.63	.85
**PC ae C36:1**	7.74	3.79	8.25	3.67	.69
**PC ae C36:2**	14.35	±4.73	13.51	8.52	.90
**PC ae C36:3**	7.05	2.68	6.68	3.34	.40
**PC ae C36:4**	18.35	10.87	16.08	7.64	.24
**PC ae C36:5**	11.86	±4.92	10.58	4.45	.52
**PC ae C38:0**	1.97	1.10	1.97	0.99	.84
**PC ae C38:1**	0.52	±0.43	0.56	0.49	.43
**PC ae C38:2**	1.93	1.10	2.01	1.16	.89
**PC ae C38:3**	3.71	2.02	3.76	1.49	.69
**PC ae C38:4**	12.90	7.00	12.77	4.24	.41
**PC ae C38:5**	17.10	8.25	16.56	6.86	.28
**PC ae C38:6**	7.56	3.39	7.25	4.37	.92
**PC ae C40:1**	1.54	±0.78	1.47	0.56	.70
**PC ae C40:2**	1.55	0.57	1.64	0.77	.47
**PC ae C40:3**	0.82	0.20	0.87	0.36	.47
**PC ae C40:4**	2.09	1.03	1.89	0.92	.14
**PC ae C40:5**	3.09	1.25	3.28	1.27	.81
**PC ae C40:6**	4.25	2.16	4.31	2.25	.79
**PC ae C42:1**	0.63	±0.30	0.56	0.32	.62
**PC ae C42:2**	0.62	0.26	0.62	0.45	.99
**PC ae C42:3**	0.85	0.31	0.90	0.47	.54
**PC ae C42:4**	0.68	0.23	0.65	0.29	.68
**PC ae C42:5**	1.84	0.65	1.67	0.59	.16
**PC ae C44:3**	0.21	0.10	0.22	0.18	.76
**PC ae C44:4**	0.33	0.10	0.31	0.17	.55
**PC ae C44:5**	1.46	0.59	1.32	0.70	.11
**PC ae C44:6**	1.09	0.43	1.04	0.52	.20
**SM (OH) C14:1**	4.94	1.73	5.20	2.30	.62

**SM (OH) C16:1**	2.59	±0.74	2.45	1.03	.77
**SM (OH) C22:1**	9.09	5.06	9.28	3.72	.71
**SM (OH) C22:2**	6.91	2.84	7.19	2.91	.74
**SM (OH) C24:1**	0.84	0.26	0.83	0.42	.75
**SM C16:0**	93.65	29.38	98.08	46.41	.75
**SM C16:1**	12.10	4.17	12.15	5.41	.87
**SM C18:0**	23.35	10.02	22.58	11.90	.80
**SM C18:1**	7.65	3.46	7.21	4.38	.87
**SM C20:2**	0.24	0.15	0.24	0.10	.93
**SM C24:0**	14.58	±4.72	14.28	6.82	.84
**SM C24:1**	38.25	14.97	42.37	17.74	.32
**SM C26:0**	0.17	±0.07	0.15	0.10	.55
**SM C26:1**	0.37	±0.15	0.40	0.20	.39
**H1**	6467.50	2202.11	6706.54	2745.04	.54
**AAA**	243.19	89.92	257.43	57.28	.39
**ADMA / Arg**	0.0053	0.0026	0.0050	0.0024	.95
**BCAA**	647.44	259.51	668.51	253.77	.30
**Cit / Arg**	0.37	0.12	0.35	0.17	.73
**Cit / Orn**	0.40	0.17	0.38	0.18	.51
**Essential AA**	1658.21	624.28	1751.19	734.60	.61
**Fisher ratio**	2.62	0.60	2.65	0.87	.51
**Glucogenic AA**	4198	1054	4181	862	.79
**Kynurenine / Trp**	0.06	0.03	0.05	0.02	.031
**Orn / Arg**	0.90	0.40	0.92	0.43	.74
**Putrescine / Orn**	0.00	0.00	0.00	0.00	.62
**Serotonin / Trp**	0.01	0.01	0.01	0.01	.95
**Total SM**	217.63	78.62	221.83	81.27	.65
**Total SM-non OH**	188.70	63.67	197.37	70.75	.72
**Total SM-OH**	24.08	10.78	24.52	10.08	.81
**Total SM-OH / Total SM-non OH**	0.13	0.03	0.13	±0.03	.85
**Tyr / Phe**	1.07	0.37	1.11	±0.19	.86

Medians and IQR are given if not otherwise indicated. **±** **-** Mean and SD are given. a - acyl; aa - diacyl; ae, acyl-alkyl.
