# Supplementary material for: Effects of RIPC on the Metabolomical Profile during Lower Limb Digital Subtraction Angiography: A Randomized Controlled Trial
Source: Metabolites. 2023 Jul 18;13(7):856. doi: 10.3390/metabo13070856 (PMC10384110; doi:10.3390/metabo13070856)
Supplement: Supplementary file 1 [file metabolites-13-00856-s001.zip › Table S3 - Significant changes in metabolite levels 24 hours after intervention between the RIPC and sham group.pdf]

Table S3. Significant changes in metabolite levels 24 hours after intervention between the RIPC and sham group.

	RIPC		SHAM				
	Δ Within Groups				Δ Between Groups *		
	**Median**	***p*-Value**	**Median**	***p*-Value**	**U-Statistic**	**Effect Sizes**	***p*-Value**
**Glutamate**	7.36	.37	-18.01	.004	874	-0.083	.011
**Taurine**	7.76	.29	-11.98	.029	911	-0.068	.022
**Asymmetric dimethyl arginine to arginine ratio**	0.000166	.46	-0.000582	.011	898.5	-0.073	.017
**LysoPC a C24:0**	-0.030	.39	-0.205	.001	929	-0.061	.030
**LysoPC a C26:1**	0.011	.79	-0.249	.001	843.5	-0.096	.006
**LysoPC a C28:0**	-0.039	.69	-0.234	.001	920.5	-0.065	.026
**PC ae C30:2**	0.0080	.51	-0.0269	.002	866	-0.087	.009
**PC aa C38:1**	0.161	.14	-0.309	.041	883.5	-0.080	.013
**PC ae C44:3**	-0.0038	.97	-0.0410	.001	907.5	-0.070	.021

* - All statistical analyses reflecting changes between the groups were conducted using the Mann-Whitney U test. Only the results that are statistically significant, following the application of the Benjamini-Hochberg method for multiple comparison correction, are provided. Changes in metabolite concentrations were calculated by subtracting the baseline measurement values from the 24-hour measurement values and are given in units of micromolar (μM). The Hodg-es-Lehmann method was used to calculate the effect sizes. LysoPC- lysophosphatidylcholine; PC- phosphatidylcholine; a- acyl; aa- diacyl; ae- acyl-alkyl.
