# Supplementary material for: Effects of RIPC on the Metabolomical Profile during Lower Limb Digital Subtraction Angiography: A Randomized Controlled Trial
Source: Metabolites. 2023 Jul 18;13(7):856. doi: 10.3390/metabo13070856 (PMC10384110; doi:10.3390/metabo13070856)
Supplement: Supplementary file 1 [file metabolites-13-00856-s001.zip › Table S4 - Correlations of significant hemodynamic, biochemical and metabolic changes.pdf]

Table S4. Correlations of significant haemodynamic, biochemical and metabolic changes.

		Glutamate		Taurine		ADMA to Arginine Ratio		LysoPC a C24:0		LysoPC a C28:0		LysoPC a C26:1		PC aa C38:1		PC ae C30:2		PC ae C44:3	
		RIPC	SHAM	RIPC	SHAM	RIPC	SHAM	RIPC	SHAM	RIPC	SHAM	RIPC	SHAM	RIPC	SHAM	RIPC	SHAM	RIPC	SHAM
**AIx**	Spearman´s rho	-0.006	-0.204	0.151	-0.267	0.069	-0.038	0.060	-0.135	0.044	-0.088	0.115	-0.049	-0.056	0.060	-0.012	-0.040	0.193	0.070
	p-value	.967	.140	.315	.051	.646	.786	.692	.332	.771	.528	.449	.725	.710	.669	.935	.773	.199	.617
**AIx@75%**	Spearman´s rho	-0.038	-0.282*	0.233	-0.247	0.108	-0.042	0.046	-0.235	0.035	-0.194	0.097	-0.174	-0.007	-0.037	0.079	-0.098	0.132	-0.029
	p-value	.802	.039	.119	.071	.475	.764	.761	.087	.819	.159	.522	.207	.966	.789	.601	.483	.383	.836
**MAP**	Spearman´s rho	-0.183	-0.113	0.036	0.020	-0.132	-0.065	0.361*	-0.081	0.290	-0.259	0.306*	-0.182	0.246	0.052	0.049	-0.224	-0.002	0.073
	p-value	.225	.414	.814	.888	.381	.642	.014	.561	.051	.058	.039	.189	.100	.709	.748	.104	.987	.600
**PWV**	Spearman´s rho	-0.013	-0.090	-0.108	-0.011	0.009	0.007	-0.138	0.069	-0.159	-0.074	-0.072	-0.035	0.058	0.122	-0.149	-0.013	-0.063	0.068
	p-value	.929	.519	.474	.935	.952	.959	.362	.619	.291	.595	.634	.800	.704	.378	.323	.925	.676	.625
**PSBP**	Spearman´s rho	-0.151	0.056	0.025	0.052	-0.043	-0.044	0.355*	0.027	0.345*	-0.162	0.402**	-0.101	0.228	0.010	0.059	-0.122	0.040	0.119
	p-value	.317	.687	.870	.709	.779	.751	.015	.844	.019	.242	.006	.469	.128	.942	.698	.380	.792	.392
**CSBP**	Spearman´s rho	-0.136	-0.039	0.043	0.026	-0.066	-0.042	0.357*	-0.024	0.333*	-0.188	0.405**	-0.119	0.186	0.068	0.054	-0.137	0.100	0.131
	p-value	.369	.777	.777	.853	.665	.762	.015	.861	.024	.173	.005	.392	.216	.625	.720	.322	.509	0.344
**Adiponectin**	Spearman´s rho	-0.338*	0.216	-0.137	-0.144	-0.316*	-0.209	0.121	0.135	0.051	0.117	0.089	0.145	-0.179	-0.065	0.220	0.094	-0.239	-0.062
	p-value	.021	.117	.365	.297	.032	.129	.424	.331	.735	.399	.557	.296	.233	.639	.142	.498	.110	.657
**Hs-Troponin-T**	Spearman´s rho	-0.020	0.020	0.197	0.100	-0.205	0.074	0.165	-0.001	0.193	-0.070	0.107	-0.073	0.242	0.011	0.178	0.092	0.100	-0.131
	p-value	.896	.883	.189	.470	.173	.594	.274	.993	.198	.614	.480	.598	.105	.937	.236	.508	.509	.346
**CK-MB**	Spearman´s rho	-0.201	-0.107	-0.152	0.112	-0.100	-0.026	0.034	-0.186	-0.138	-0.235	-0.115	-0.126	-0.169	-0.090	-0.099	-0.072	-0.193	-0.039
	p-value	.181	.442	.313	.422	.509	.849	.825	.178	.362	.087	.448	.364	.262	.519	.514	.605	.199	.777
**NT-proBNP**	Spearman´s rho	-0.089	-0.080	-0.085	-0.042	-0.067	-0.171	0.018	-0.003	-0.038	-0.085	0.033	-0.014	0.092	-0.155	-0.028	0.022	-0.056	0.123
	p-value	.559	.564	.575	.764	.657	.217	.908	.983	.803	.543	.828	.919	.542	.263	.852	.872	.711	.374
**Creatinine**	Spearman´s rho	-.303*	-0.213	-0.191	-0.168	0.038	-0.075	-0.017	0.080	-0.035	0.202	-0.117	0.175	-0.193	0.180	-0.154	0.047	0.002	-0.107
	p-value	.041	.122	.203	.225	.804	.589	.912	.564	.820	.143	.439	.206	.200	.194	.306	.735	.987	.442
**eGFR**	Spearman´s rho	0.261	0.199	0.224	0.155	0.045	0.076	0.002	-0.117	-0.001	-0.245	0.083	-0.205	0.114	-0.194	0.081	-0.090	-0.028	0.076
	p-value	.080	.150	.135	.264	.768	.586	.989	.398	.996	.074	.582	.136	.449	.160	.592	.516	.852	.583
**Urea**	Spearman´s rho	-0.056	-0.034	0.069	-0.102	0.088	0.232	0.286	-0.130	0.228	-0.063	0.162	-0.078	0.084	0.196	0.201	0.008	0.119	-0.122
	p-value	.713	.809	.650	.464	.563	.091	.054	.347	.128	.650	.283	.575	.580	.156	.181	.952	.432	.379

Correlation coefficients of Spearman´s rho are represented. AIx- augmentation index; AIx@75%- heart rate corrected augmentation index; MAP-mean arterial pressure; PWV- pulse wave velocity; PSBP- peripheral systolic blood pressure; CSBP- central systolic blood pressure; hs-Troponin-T – high sensitivity troponin T; CK-MB – creatinine kinase MB isoenzyme; NT-proBNP - N-terminal pro b-type natriuretic peptide; eGFR- estimated glomerular filtration rate; ADMA – asymmetric dimethyl arginine; LysoPC- lysophosphatidylcholine; PC- phosphatidylcholine; a- acyl; aa- diacyl; ae- acyl-alkyl; ** - correlation is significant at the .01 level (2-tailed); * - correlation is significant at the .05 level (2-tailed).
